# Polystyrene Biodegradation by *Tenebrio molitor* Larvae: Identification of Generated Substances Using a GC-MS Untargeted Screening Method

**DOI:** 10.3390/polym13010017

**Published:** 2020-12-23

**Authors:** Emmanouil Tsochatzis, Joao Alberto Lopes, Helen Gika, Georgios Theodoridis

**Affiliations:** 1 Department of Food Science, Centre of Innovative Food Research (iFood), Aarhus University, Agro Food Park 48, 8200 Aarhus N, Denmark; 2 European Commission, Joint Research Centre (JRC), 2440 Geel, Belgium; Joao-Filipe.ALBERTO-LOPES@ec.europa.eu (J.A.L.); gtheodor@chem.auth.gr (G.T.); 3 Department of Medicine, Aristotle University of Thessaloniki, 54124 Thessaloniki, Greece; gkikae@auth.gr; 4 Department of Chemistry, Aristotle University of Thessaloniki, 54124 Thessaloniki, Greece; 5 FoodOmicsGR Research Infrastructure, AUTh Node, Center for Interdisciplinary Research and Innovation (CIRI-AUTH), Balkan Center B1.4, 10th Km Thessaloniki-Thermi Rd, P.O. Box 8318, 57001 Thessaloniki, Greece

**Keywords:** plastics biodegradation and biorecycling, GC-MS analysis, chemical compounds formed during bio-degradation, insects assisted biodegradation of polystyrene

## Abstract

A GC-MS method has been applied to screen and evaluate the generation of chemical compounds during the biodegradation of polystyrene (PS) with *Tenebrio molitor* larvae. Several resulting compounds have been identified, including trimers 2,4,6-triphenyl-1-hexene and 1,3,5-triphenylcyclohexane, the volatiles acetophenone and cumyl alcohol, and 2,4-di-tert butylphenol, a non-intentionally added substance (NIAS) present in the plastic material. The PS monomers styrene and α-methyl styrene were also identified in the extracts. Bioactive molecules present in the biomass of the studied insects were identified, such as the free fatty acids myristic, palmitic, and oleic acid. Undecanoic acid was also found, but in lower mass fractions. Finally, biochemically formatted amides resulting from their respective fatty acids were identified, namely tetradecanamide, hexadecanamide and oleamide. The formation of all these substances seems to suggest enzymatic and biochemical activity occurring during the biodegradation of PS, and their amounts varied throughout the experience. The overall degradation rate of PS resulted in a 13% rate, which highlights the potential of biorecycling using these insects.

## 1. Introduction

Plastic packaging is associated with raised concerns about the potential harm to the environment and human health [1]. Polystyrene (PS) is a thermoplastic polymer considered the third most important for consumers and industry. It is used extensively to produce food contact materials (FCMs) due to its low cost, durability and mechanical strength [2]. Large production amounts, associated to low recycling rates, have resulted in the accumulation of PS and other plastics in the environment, further risking the survival of wildlife and human health [3]. 

There is an increasing amount of interest and research conducted on the biodegradation of plastic based on activity of bacteria and fungi, as these were found to be capable of degrading plastic materials. Some studies already reported the potential degradation of PS by using different insects: mealworms (*Tenebrio molitor*) [4,5,6], superworms (*Zophobas morio*) [7] and waxworms (*Galleria mellonella*) [3]. Furthermore, bio-recycling by insects was reported also for other polymers like polyethylene (PE) [8,9]. However, the existing knowledge is not yet sufficient to allow the full evaluation of the process in relation to the degradation of PS. This is particularly important for the potential generated substances due to the insects’ activity, especially regarding the formation/release of residual monomers, oligomers or other non-intentionally added substances (NIAS).

Recently the European Food Safety Authority (EFSA) re-evaluated the safety of styrene (FCM No. 193 in Reg. (EU) No. 10/2011) in plastic FCMs. This reevaluation followed a report from International Agency for Research on Cancer (IARC) that highlighted styrene as probably carcinogenic to humans. The EFSA concluded that styrene’s potential genotoxicity could not be excluded [10,11]. Thus, it becomes critical to take in consideration the formation of styrene, α-methyl styrene and oligomers resulting from PS recycling or bio-recycling, as to avoid environmental pollution or exposure to such potentially genotoxic compounds [1].

Styrene and α-methyl styrene are EU regulated substances allowed to be used in the production of polymers [12]. However, PS oligomers are considered as NIAS, and can be formed during the production processes or due to decomposition reaction [12]. NIAS can end up in the polymeric material and migrate into the food. Hence, residual PS monomers and oligomers can be considered of high importance, and their study is crucial for establishing health safety and environmental impact [13,14]. 

Analysis of styrene, α-methyl styrene and related oligomers have been performed in the context of multianalyte GC-MS methods [15]. Dedicated targeted GC-MS methods for those substances have also been reported [1]. Regarding untargeted screening of insects’ frass, some qualitative GC-MS methods also exist, but no quantification of the target analytes was performed [3,5,6].

The scope of this research was to perform a preliminary evaluation of the biodegradation rate of PS by using it as feed of *Tenebrio molitor* larvae. It aimed at identifying and quantifying the most relevant substances (degradation products, PS oligomers or other bioactive compounds) resulting from the insect’s metabolic process. This evaluation allowed us to understand if compounds of significant added value are being produced that later on can be exploited. To the best of the authors’ knowledge, it is the first time this research was conducted, and it represents a first step in assessing the PS bio-recycling capacity of the used insects. 

## 2. Materials and Methods 

### 2.1. Chemicals

Dichloromethane (DCM; CAS: 75-09-2) of Chromasolv grade purity was obtained from Sigma Aldrich (Steinheim, Germany). PTFE 17 mm. 0.2 μm membrane filters were supplied from Sigma Aldrich (Steinheim, Germany). Styrene (≥96%), α-methyl styrene (99%), acetophenone (≥98%), α,α-dimethyl benzene methanol (cumyl alcohol) (≥97%), ethyl linoleate (≥99%), ethyl hexadecanoate (≥99%), 2,4-di-tert butyl phenol (DTBP) (99%), methyl-9,12-octadecadienoate (≥98%), tetradecanamide (≥96%), and oleamide (≥99%) were also purchased from Sigma Aldrich (Steinheim, Germany). Hexadecanamide (95%) was purchased from TCI Chemicals (Tokyo, Japan). The PS oligomers 2,4,6-triphenyl-1-hexene and 1,3,5-triphenylcyclohexane (mixture of isomers) were purchased from FUJIFILM Wako Chemicals Europe GmbH (Milano, Italy).

### 2.2. Polystyrene Samples

The expanded PS (EPS) foamed plaques (density 0.010 g/cm^3^) were bought at a local do it yourself (DIY) store. A verification of the PS plaques was performed by attenuated total reflection–Fourier transform infrared (ATR-FTIR) spectroscopy and differential scanning calorimetry (DSC). Recorded spectra and thermograms were compared with the in-house database and confirmed the PS nature of the samples (matching FTIR spectra and a glass transition of 100 °C). However, the Mn of the studied type of PS was not possible to confirm, although it is expected to be around Mn = 100,000, the most common type existing in the market. 

### 2.3. Biodegradation of Polystyrene

The followed procedure was based in the one reported by Lou et al. [3]. The *Tenebrio molitor* larvae (average weight 72–80 mg/larvae) were purchased from Huiyude Co. (Tianjin, China) and were starved for 36 h before the experiment to prevent any effect of previously eaten feed [3]. The larvae were then fed with a single PS diet, and each experiment was done in triplicate. There were 100 larvae in each of the three 15 × 15 × 5 cm^3^ sized containers (per treatment), along with 1.5 g of PS plastic cut into 1 cm cubes. The containers were placed in an incubator maintained at 27.0 ± 0.5 °C and with humidity of 75 ± 5%. The experiments were performed in triplicates. Fifty larvae were extracted and analyzed before the biodegradation experiment to set up control (blank) samples, and then larvae, from a single box representing each sampling day, were fed and collected (day 1, 3 and 7, after the beginning of the bio-recycling) and further analyzed. The duration of the experiment was seven days.

### 2.4. Lyophilzation

The collected *Tenebrio molitor* larvae were blended for 2 min in a domestic blender after being weighted. Subsequently they were lyophilized by a Gamma 1–20 freeze-dryer (Osterode am Harz, Germany) at −20 °C and 1.030 mbar. Shelf temperature of the freeze-dryer was adjusted to 25 °C. The resulting powder samples were then stored at −20 °C until analysis, following the conditions from Tsochatzis et al. [16].

### 2.5. Solid-Liquid Extraction

For the extraction, a method previously reported by our group [15] was slightly modified. Briefly, a lyophilized sample of 0.5 g of the collected insects’ larvae was placed in a glass tube and extracted twice, with 1.0 mL of dichloromethane (DCM). The tube was vortexed for 1 min and sonicated for 2 h, at 25 °C, followed by centrifugation at 20 °C and 2500 rpm (1280× *g*) for 5 min. Finally, samples were filtered with PTFE 0.22 mm filters and filled to a final volume of 2.0 mL with DCM. A 1 μL aliquot was then injected in the GC system.

### 2.6. GC-MS Analysis

Chromatographic analyses were performed with a GC system Agilent Technologies 7890B GC (Waldbronn, Germany) equipped with a triple quadrupole mass detector (Agilent Technologies 7010-MS), operated at constant helium flow rate (1.5 mL min^−1^). The chromatographic column (HP-Innowax, 30 m × 250 μm, 0.25 μm) was supplied by Agilent Technologies Inc. (Santa Clara, CA, USA). The analysis was performed using a split/split-less injector at 300 °C in splitless mode, with a purge flow of 50 mL min^−1^ and a purge time of 1.5 min, using a single taper liner. The injection volume was 1 μL. The oven program was the follow: initial temperature of 40 °C for 3 min, ramp (25 °C min^−1^) up to 265 °C (isothermal of 12.5 min), ramp (10 °C min^−1^) up to 270 °C (isothermal of 2 min). The total run time was 27 min, including a solvent delay time of 4.5 min, applying Electron Impact Ionization was operated at 70 eV. Mass analysis and detection was performed using full scan 40 to 700 amu. The resulting chromatograms were processed and deconvoluted using MassHunter software (Agilent Technologies Inc., Santa Clara, CA, USA) while for the identification NIST11 library was used. Further spectral and analytical data for the PS oligomers were based on previous reported research work [1].

### 2.7. Differential Scanning Calorimetry (DSC)

DSC analyses were performed with a TA Instruments Model Q100 (Newcastle, DE, USA) equipped with an autosampler. The temperature control program consisted of heating from 0 °C to 200 °C at 10 °C/min (1st cycle), cooling to 0 °C at 10 °C/min (2nd cycle) and heating back to 200 °C at 10 °C/min. The heating scans were performed with the sample under a constant flow of nitrogen at 50 mL min^−1^.

### 2.8. Fourier-Transform Infrared Spectroscopy (FTIR)

All spectra were acquired with a diamond crystal attenuated total reflectance (ATR) FTIR spectrometer (Perkin Elmer Spectrum 2000, Waltham, MA, USA). Spectra were acquired in the scan range 4000.00–530.00 cm^−1^, with a resolution of 4.00 cm^−1^ and a total of 8 scans for each sample that were averaged to eliminate background noise. Samples were analyzed without any preparation and the obtained spectra were compared with a spectral database.

### 2.9. Quantification of Identified Compounds

For the quantification of the identified compounds an in-house GC-TIC-MS validated method was used. This method was developed using analytical standards and the standard addition method for the calibration, following the analytical conditions reported in Section 2.6. The linearity was evaluated by the linear regression coefficient (R^2^). Limit of detections (LOD) and limits of quantification (LOQ) were evaluated from the signal-to-noise (S/N) ratio. Mean values and standard deviation of the S/N were retrieved from 5 chromatograms at the lowest calibration levels. The LOD was calculated as 3 times the S/N, while the LOQ was calculated as 3 times the LOD [17,18]. Precision and trueness were assessed only for short-term repeatability, with three replicates of fortified samples during the same day. 

### 2.10. Data Processing and Statistical Analysis

Data were processed using the Agilent Technologies Masshunter 10.0 software (Agilent Technologies Inc., Santa Clara, CA, USA). Regression analysis and statistics were performed using Microsoft Excel, and further statistical analysis, such as one-way analysis of variance (ANOVA), followed by Tukey comparison test in all cases, has been performed with Minitab 18.0 statistical software (Minitab Inc., State College, PA, USA). 

## 3. Results

### 3.1. Untargeted Screening and Identification of Chemical Compounds

**Figure 1:**
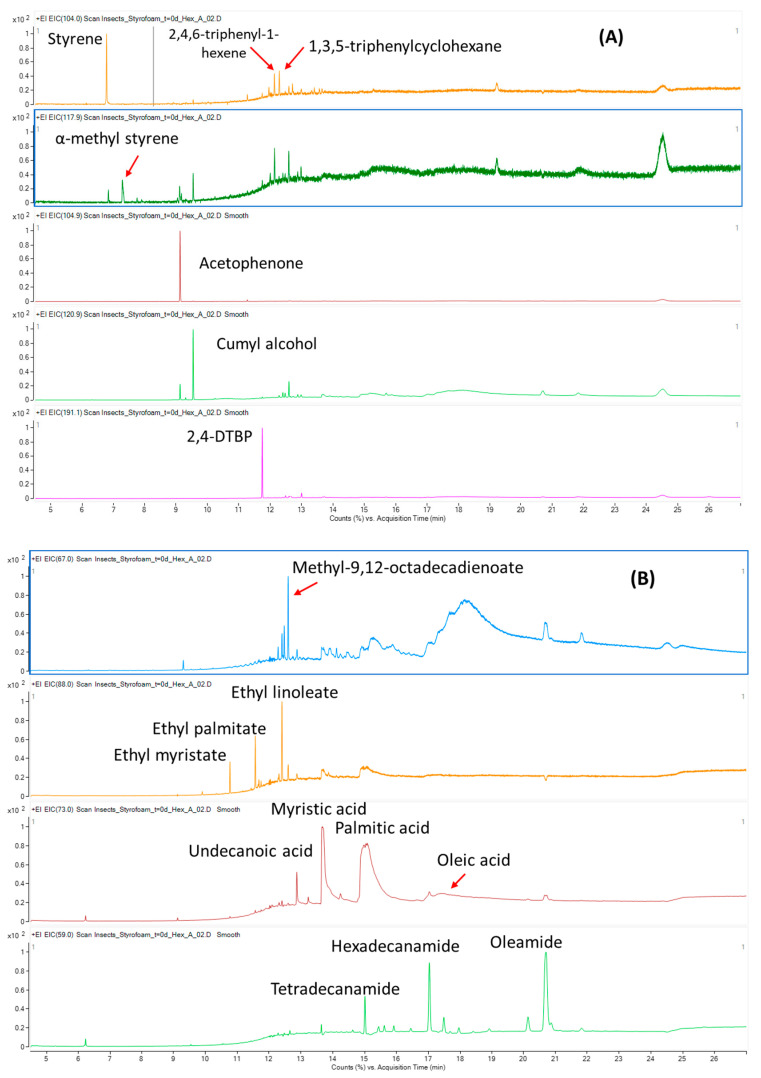
GC-MS extracted ion chromatogram (EIC) of lyophilized biomass of *Tenebrio molitor* larvae, after 3 days of biodegradation of PS, of (**A**) plastic related compounds (monomers, oligomers and NIAS); (**B**) bioactive compounds (fatty acids, fatty acid esters and respective amides).

The selection of the extraction solvent was based on previous studies, where it was shown that DCM is a good extracting solvent for a majority of the FCM substances, NIAS and PS monomers and oligomers [1,15]. The results indicate that there are several substances existing within the insects’ biomass, apart from the residual monomers, oligomers or plastic additives. The identified compounds are reported in Table 1, with Figure 1 presenting the extracted ion chromatograms (EIC) of one of the extracted larvae biomass samples (day 3), from the Total Ion Chromatogram (TIC; Figure S1).

Performance characteristics of the used in-house analytical method are presented as (Supplementary Material Table S1). Briefly, linearity was above 0.99 for all analytes and LODs ranged from 0.009 mg kg^−1^ (cumyl alcohol) up to 0.020 mg kg^−1^ for 9-octadecenamide. Relative standard deviations (RSD, %) were below 16% and recoveries ranged from 84.0% (tetradecanamide, high mass fraction) to 104.9% (ethyl hexadecanoate; low mass fraction). 

Untargeted screening of the extracted larvae biomass provided a number of interesting peaks which were processed further by studying the NIST spectral library. Identified substances were further assessed and confirmed based on their respective and representative m/z and a similarity index higher than 80%. Furthermore, for PS monomers and oligomers existing spectral data were used [1,15]. Finally, the preliminary identification of substances was confirmed by injecting respective analytical standards for every single suspect (Table 1). 

**Figure 2:**
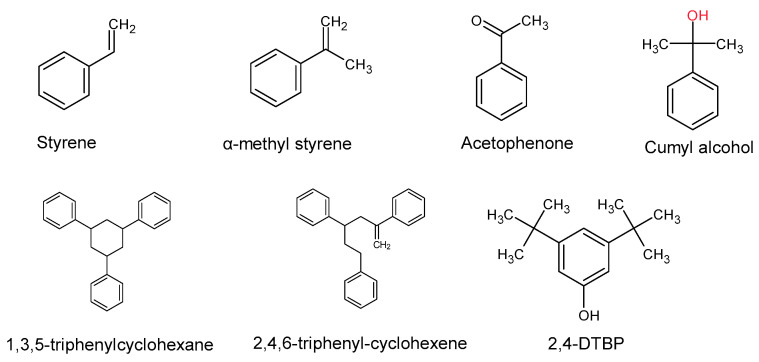
Chemical structures of seven of the identified PS monomers and oligomers and a non-intentionally added substance (NIAS) (2,4-DTBP) resulting from biodegradation of PS by *Tenebrio molitor*.

Apart from the identified PS monomers (styrene, α-methyl styrene), additional PS oligomers were identified: 2,4,6-triphenyl-1-hexene and 1,3,5-triphenylcyclohexane, both trimers. Their structures are presented in Figure 2. 

Some fatty acids existing in the extracts were also identified: (1) the saturated fatty acids (SFA) undecanoic acid (undecylic acid), tetradecanoic acid (myristic acid) and hexadecanoic acid (palmitic acid), and (2) the unsaturated (UFA) oleic acid. All these acids were present in relatively high amounts, with the exception being undecylic acid, found at traces levels.

We have additionally identified methyl-9,12-octadecadienoate (methyl linoleate), ethyl-9,12-octadecadienoate (ethyl linoleate) and ethyl hexadecanoate (ethyl palmitate), which are esters of the above-mentioned fatty acids. Finally, amides directly related to the identified fatty acids were also found: tetradecanamide, hexadecanamide, and 9-octadecenamide (oleamide).

**Figure 3:**
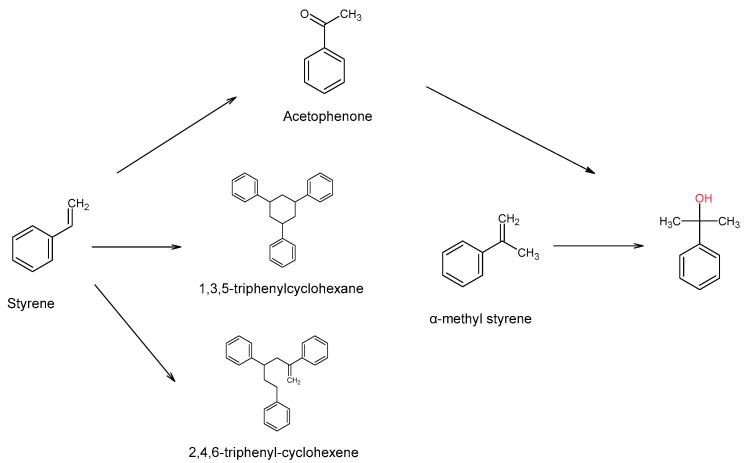
Identified PS degradation compounds and PS oligomers resulting from the biorecycling.

A representative scheme of the identified PS monomers and substances resulting from their degradation/reaction is presented in Figure 3.

### 3.2. Degradation Rate

**Figure 4:**
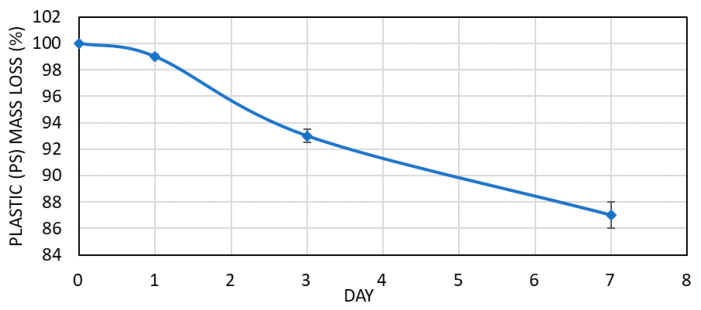
Plastic consumption by *Tenebrio molitor* in the plastic-fed diets over 7-days.

The mass differences originated by the degradation of the PS within the time period of the experiment were registered (Figure 4). Hence, mass balance showed that from the initial amounts of PS a significant statistical degradation rate (*p* < 0.05) existed among the different sampling days, pointing at a non-linear degradation. The total degradation rate at the end of the experiment (7 days) was ca. 13%, which can be considered as sufficient and in line with previously reported studies [7,19]. A longer experiment time could eventually lead to higher degradation rates. It must be noted that during the experiment no assessment of insects’ survival rate was performed

**Figure 5:**
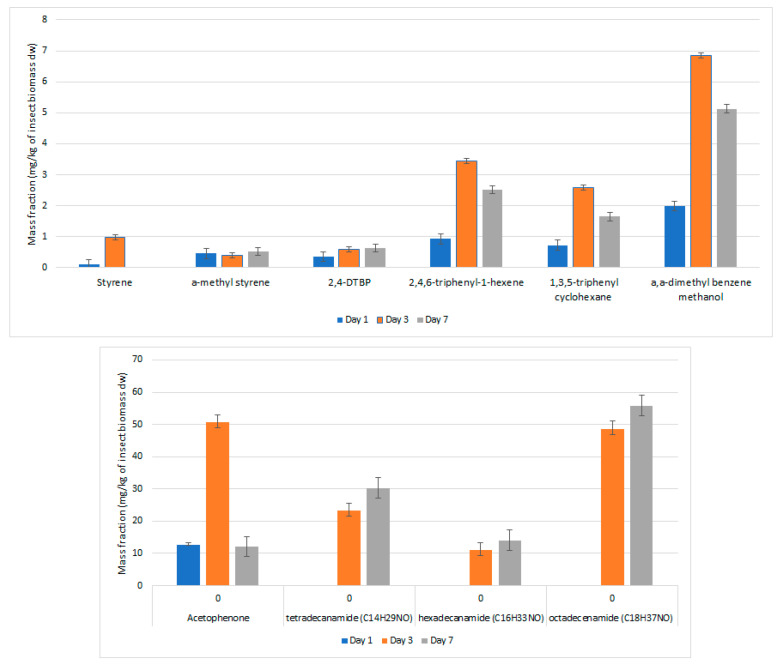
Fate and release assessment of chemical from PS biodegradation by *Tenebrio molitor* larvae.

The formation rate of the identified substances during the biodegradation experiment is presented graphically in Figure 5. A simple one-way ANOVA analysis with Tukey test comparison highlighted significant differences in all the identified substances, except in the case of α-methyl styrene and 2,4-DTBP from day 3 to day 7.

## 4. Discussion

### 4.1. Analytical Method and Identified Compounds 

The applied analytical method was considered fit-for-purpose for this study, taking into consideration its existing thresholds and limitations [18,20]. The selection of the extraction solvent was based on previous studies, where it was shown that DCM is good extracting solvent for a majority of the FCM substances, PS monomers and oligomers, and NIAS [1,15]. Moreover, although a polar column (Innowax) has been used, the peak quality for the fatty acids was low, with their identification being based on NIST library and injection of standards. The results of day 1 indicated the presence of the PS monomers styrene and α-methyl styrene, substances that were not found in the extracts of the biomasses of the larvae that were not fed with PS (day 0). Their formation was expected, as it indicates that a degradation of the polymer occurs inside insects’ gut, due to enzyme-based microbiological activity [7]. Furthermore, we identified acetophenone and cumyl alcohol. The origin of the former can be either the result of the oxidation of styrene [21] or a degradation product from PS production. However, no residues of acetophenone existed before the biodegradation, which seems to support the assumption that it is a bio-oxidation product from PS/styrene due to enzymatic activity [21,22]. Acetophenone has been reported to act as substrate for the formation of cumyl alcohol, either via oxidation [23] or by enzymatic addition (hydratases) to double C-C bonds [24], which can occur in the case of α-methyl styrene (substitution). However, the amounts of α-methyl styrene were significantly lower when compared to styrene, which may indicate that the latter is responsible for its formation. Nevertheless, acetophenone formation could come from both monomers, as presented in Figure 4. 

As already reported by Tsochatzis et al. [1], a significant amount of low molecular weight residual monomers and oligomers can be present in a plastic material. The PS monomeric and oligomeric content in packaging can be considered of importance and with potential health related impact [1]. That is the case of styrene, which use in FCMs has been recently revaluated by EFSA [10]. Similar concerns may also exist for the cases of styrene dimers and trimers, as reported recently [1]. 

In this study the formation of two PS trimers 2,4,6-triphenyl-1-hexene and 1,3,5-triphenylcyclohexane have been reported [1]. Their concentration in the biomasses extracts gradually increased from day 0 to day 7, which can be attributed to the bio-degradation of the PS plastic by the *Tenebrio molitor* larvae (Figure 2 and Figure 3). 

Several free fatty acids (FFA) were also identified, both saturated (myristic, palmitic, undecanoic acid) and unsaturated (oleic acid). Although the chromatographic response suggests the existence of these acids in high amounts in the samples, their exact quantification was not possible. Quantification of these substances would require extensive sample derivatization, which would fall out of the scope of this work. The formation of FFAs in identical experiments has been reported by Kong et al. In that work, during the biodegradation of plastics with the use of *Galleria mellonella* long-chain FFA were produced from long-chain hydrocarbon waxes. This formation happened without microbial assistance, which could point to results of enzymatic activity only [25]. 

Our results are also consistent with what was reported by Lou et al., although they indicated that in the case of biodegradation of plastics by insects, the formation of C=O and C−O containing functional groups and long chain fatty acids, is indicative of metabolic intermediates of plastics depolymerization and biodegradation [3].

The presence of tetradecanamide, hexadecanamide, and 9-octadecenamide (oleamide) in the larvae biomass extracts is rather intriguing, especially when considering that they occur concomitantly with their acid equivalents. Amide bond formation serves as a fundamental reaction in chemistry and in nature. The most abundant biological macromolecules linked with amide bonds are peptides and proteins, resulting by a ribosome system in living organisms. Biocatalytic amide bond formation could be biochemically achievable through several potential enzymatic/chem-enzymatic methods, such as aminolysis reaction by hydrolases, peptide formation between amino acid esters and amino acids by acyltransferases, ligands formation with two or more amino acids (unprotected) by ATP-dependent ligases, nucleophilic substitution by transpeptidases, and hydration of nitriles to amides by nitrile hydratases [26]. Furthermore, non-ribosomal peptide synthetase (NRPS) may provide another pathway. For the latter, this could be realized by ATP-dependent enzymes that generate acylphosphate intermediates or through ATP independent transacylation [27]. Nevertheless, the enzymatic formation of an amide bond is therefore a particularly interesting platform for engineering the synthesis of structurally diverse natural and unnatural ABC molecules for applications in drug discovery and molecular design.

2,4-Di-tert-butylphenol (2,4-DTBP) is a NIAS if found in FCMs, as it is not EU regulated. It originates from the degradation of a common antioxidant used as additive in FCMs, Irgafos 168 (tris (2,4-di-tert-butylphenyl) phosphate). Less reported data is available for 2,4-DTBP, but its toxicity on rats has been studied, revealing no-observed adverse- effect levels (NOAEL). The identification of 2,4-DTBP and Irgafos 168 is frequent when analyzing FCM-related matrices [1]. However, the latter was not detected by our analytical system, probably due to the use of a polar analytical column (Innowax) and its high molar mass (647 Da). Thus, we observe that the 2,4-DTBP amounts increase significantly at the beginning of the biodegradation and they reach a “plateau” after 7 days. No results are available for the fate of this compound, especially within a biochemical (biodegradation) process. 

Our results are also in accordance with reported data by Son et al. regarding the predominant FFA within insects (myristic, palmitic and for oleic) [28]. Lou et al. analyzed the frass of PS-fed larvae and also identified long chain FFAs, such as oleic acid (C_18_H_34_O_2_), octadecanoic acid (C_18_H_36_O_2_) and *n*-hexadecanoic acid (C_16_H_32_O_2_), as well as the PS trimer (1,3,5,-triphenyl-cyclohexane), findings fully aligned with the results of this work [3]. Identifying them in this work would have required the use of a dedicated methyl-esterification method (fatty acid methyl esters; FAME) [29]. However, although interesting, it was not the main scope and focus of the current work and therefore it has not been examined further. However, the presence of long chain FFAs and the decrease of the amounts of more complex long chain carboxylic acid esters structures indicated digestion and biodegradation of PS, as reported by Lou et al. [3]. The existence of significant amounts of PS monomers and oligomers, while it shall not be underestimated the enzymatic activity, as observed in the case of amide bio-transformation, also hints at that possibility

### 4.2. Release and Fate of the Identified Chemical Compounds

The analysis of extracts of insects on day 0 did not reveal significant amounts of styrene and α-methyl styrene nor any oligomers. From day 1, the volatiles PS oligomers 2,4,6,-triphenyl -1-hexene and 1,3,5-triphenyl cyclohexane were already being formed, while also styrene and α-methyl styrene were generated. It is worthy to notice that for many other polymers, such as in the case of polyethylene terephthalate (PET), the trimer has been described as the most abundant structure [30,31]. 2,4-DTBP was also identified. A simple one-way ANOVA analysis, among the observed concentration revealed significant differences in all the identified substances, except in the case of α-methyl styrene and 2,4-DTBP, from Day 3 to Day 7. However, for 2,4-DTBP, an initial statistical increase from Day 0 to Day 3 was observed, leading to a plateau at Day 7 (gradual release from the polymer degradation). All the aforementioned effects could be explained by a limited enzymatic activity for these molecules.

The degradation rate (Figure 4) showed a non-linear behavior correlated with a statistically significant degradation rate among the different sampling days, pointing at the presence of enzymatic activities that typically require some time to take place. The latter effect can be connected also to the degradation of styrene, styrene oligomers, acetophenone and cumyl alcohol. In their case, an initial increase from day 0 to day 3 led to a decrease at day 7, something that can be explained from enzymatic activity and conversion of the ingested chemicals into CO_2_, as it has been already reported [5,19,28].

However, it is interesting to highlight that the styrene amounts increased from day 1 to day 3, followed subsequently by a decrease to non-detectable, indicating an absence of formation of that monomer between those days. In relation to the latter, we observe that α-methyl styrene is generated from day 1 and retains a ‘’plateau’’ until day 7 (Figure 5). The same plateau seems to be reached also by 2,4-DTBP, though in lower amounts. Interestingly enough, an identical behavior for acetophenone and cumyl alcohol (α,α-dimethyl benzene methanol) was observed. These compounds had an initial increase from day 1 to day 3, followed by a significant decrease from day 3 to day 7, though not as severe as in the case of acetophenone. The latter can be tentatively explained by the high volatility of acetophenone. As a concluding remark, regarding the groups of monomers and oligomers/NIAS, we observe that styrene is degraded rather fast, together with some volatile PS oligomers, while the other compounds are reaching a threshold and remaining in relatively constant mass fraction levels. The decrease observed from day 3 to day 7 can be explained by the transformation of the ingested substances to CO_2_. It has been already reported that there is a conversion ranging from 35% up to 50% of the ingested polymers into a putative gas fraction (CO_2_) [5,19,28].

Finally, and concerning the remaining bioactive molecules identified, an interesting effect was noted: the formation of amides from their respective FAs. All three identified amides (tetradecanamide, hedecanamide, oleamide) showed the same trend during the experiment: non-existing before day 3, and with a steady increase in their mass fractions up to day 7. A potential explanation can be the existing enzymatic activity, not that intense at the beginning due to temperature effects (27 °C) and humidity conditions (75% RH), that might have not been the optimal ones for this particular enzymatic activity. In addition, the formation of potential secondary or intermediate metabolites might have influenced the intensity of these phenomena.

## 5. Conclusions

This work highlighted the formation and the generation of chemical compounds during biodegradation of PS with insects’ larvae. The process was efficient, presenting a sufficient degradation rate/plastic mass loss. This degradation rate was assessed in relation to the formation or generation of compounds due to the occurring biochemical process, by applying a GC-MS based untargeted screening. Thus, several compounds have been identified including PS monomers (styrene, α-methyl styrene), PS oligomers (mostly trimers) and NIAS, either related to the nature of plastic material (acetophenone, cumyl alcohol) or due to additional plastic additives present in the initial materials (2,4-DTBP). Furthermore, several bioactive components have been identified such as FFAs (myristic, palmitic, oleic) and respective esters. Even more important was the identification of the FFA’s correspondent amides, formed after a certain period of time most probably due to the enzymatic transformationof the former.

The results of this work illustrate the existing potential in the degradation of some polymers by using them as feedstock for insects. Moreover, the observed production of some bioactive components could also be a source of obtaining interesting and valuable byproducts during the biodegradation of plastics, in what could be considered as a bio-recycling process.

## Figures and Tables

**Table 1:** Identified compounds in the extract of the biomass resulting from biodegradation of polystyrene (PS) from *Tenebrio molitor*.

Analyte	t_R_	*m*/*z*	Molecular Formula	Molecular Mass (Da)
Styrene	6.783	104.0	C_8_H_8_	104.15
α-Methyl styrene	7.291	117.9	C_9_H_10_	118.18
Acetophenone	9.127	104.9	C_8_H_8_O	120.15
α,α-Dimethyl benzene methanol (cumyl alcohol)	9.544	120.9	C_9_H_12_O	136.19
Ethyl myristate	10.770	88.0	C_16_H_32_O_2_	256.42
Ethyl palmitate	11.562	88.0	C_18_H_36_O_2_	284.48
Ethyl linoleate	12.475	67.0	C_20_H_36_O_2_	308.50
2,4-Di-tert butyl phenol (DTBP)	11.750	191.1	C_14_H_22_O	206.32
2,4,6-Triphenyl-1-hexene	12.399	117.1	C_24_H_24_	312.45
Methyl-9,12-octadecadienoate	12.775	67.0	C_19_H_34_O_2_	294.47
1,3,5-Triphenylcyclohexane	12.595	117.1	C_24_H_24_	312.45
Tetradecanamide	15.013	59.0	C_14_H_29_NO	227.39
Hexadecanamide	17.038	59.0	C_16_H_33_NO	255.44
9-Octadecenamide (oleamide)	20.691	59.0	C_18_H_35_NO	281.48
Undecanoic acid (undecylic acid)	12.815	73.0	C_11_H_22_O_2_	186.29
Tetradecanoic acid (myristic acid)	13.360	73.0	C_14_H_28_O_2_	228.37
Hexadecanoic acid (palmitic acid)	15.022	73.0	C_16_H_32_O_2_	256.42
Oleic acid	17.326	41.0	C_18_H_34_O_2_	282.46

## Supplementary Materials

The following are available online at https://www.mdpi.com/2073-4360/13/1/17/s1, Figure S1: Total Ion chromatogram (TIC) from untargeted GC-EIC-MS analysis of insects’ biomass during biodegradation of polystyrene (day 3), Table S1: Analytical features together with precision and accuracy results of the validated analytical method.
