# Supplementary material for: Polystyrene Biodegradation by Tenebrio molitor Larvae: Identification of Generated Substances Using a GC-MS Untargeted Screening Method
Source: Polymers (Basel). 2020 Dec 23;13(1):17. doi: 10.3390/polym13010017 (PMC7793077; doi:10.3390/polym13010017)
Supplement: Supplementary file 1 [file polymers-13-00017-s001.pdf]

polymers

Article

Supplementary material

Polystyrene Biodegradation by *Tenebrio molitor* Larvae: Identification of Generated Substances Using a GC-MS Untargeted Screening Method

Emmanouil Tsochatzis [1,*], Joao Alberto Lopes [2], Helen Gika [3,4,5] and Georgios Theodoridis [2]

[1] Department of Food Science, Centre of Innovative Food Research (iFood), Aarhus University, Agro Food Park 48, 8200 Aarhus N, Denmark
[2] European Commission, Joint Research Centre (JRC), 2440,Geel, Belgium; Joao-Filipe.ALBERTO-LOPES@ec.europa.eu (J.A.L.); gtheodor@chem.auth.gr (G.T.)
[3] Department of Medicine, Aristotle University of Thessaloniki, 54124 Thessaloniki, Greece; gkikae@auth.gr
[4] Department of Chemistry, Aristotle University of Thessaloniki, 54124 Thessaloniki, Greece
[5] FoodOmicsGR Research Infrastructure, AUTh Node, Center for Interdisciplinary Research and Innovation (CIRI-AUTH), Balkan Center B1.4, 10th Km Thessaloniki-Thermi Rd, P.O. Box 8318, 57001 Thessaloniki, Greece
* Correspondence: Emmanouil.tsochatzis@foodau.dk; Tel.: +45-4189-3130

Citation: Tsochatzis, E.; Lopes, J.A.; Gika, H.; Theodoridis, G. Polystyrene Biodegradation by *Tenebrio molitor* Larvae: Identification of Generated Substances Using a GC-MS Untargeted Screening Method. *Polymers* **2020**, *13*, 17. https://doi.org/10.3390/polym13010017

Received: 8 December 2020
Accepted: 21 December 2020
Published: 23 December 2020

Publisher's Note: MDPI stays neutral with regard to jurisdictional claims in published maps and institutional affiliations.

Copyright: © 2020 by the authors. Licensee MDPI, Basel, Switzerland. This article is an open access article distributed under the terms and conditions of the Creative Commons Attribution (CC BY) license (http://creativecommons.org/licenses/by/4.0/).

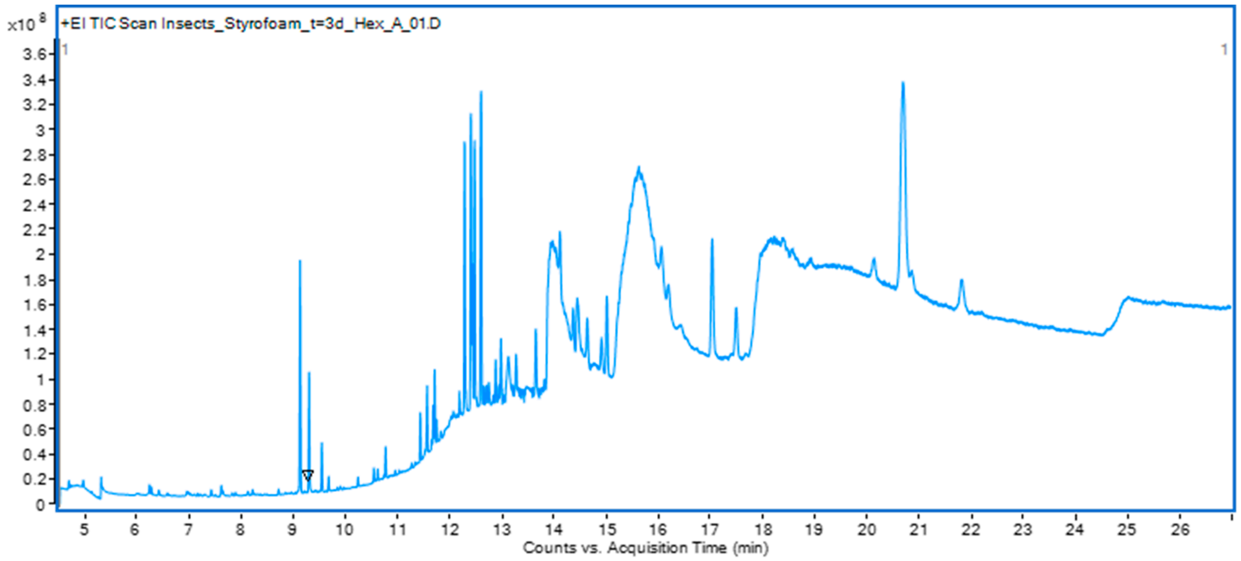

Figure S1. Total Ion chromatogram (TIC) from untargeted GC-EIC-MS analysis of insects biomass during biodegradation of polystyrene (day 3).

Table S1. Analytical features together with precision and accuracy results of the validated analytical method.

Analyte	Added (mg kg-1*)	Linear regression coefficient (R^2)	LOD (mg kg-1*)	LOQ (mg kg-1*)	RSD (%)	Recovery[a] (%)
Styrene	40.0<br>500.0	0.99	0.012	0.030	9.3<br>2.9	94.7<br>96.9
α-methyl styrene	40.0<br>500.0	0.991	0.011	0.032	15.2<br>4.5	100.3<br>96.7
Acetophenone	40.0<br>300.0	0.990	0.012	0.030	8.3<br>6.6	94.5<br>96.4
Cumyl alcohol	40.0<br>300.0	0.993	0.009	0.027	9.6<br>12.5	98.4<br>96.7
Ethyl linoleate	40.0<br>300.0	0.990	0.011	0.026	2.1<br>8.9	96.6<br>100.9
Ethyl hexadecanoate	40.0<br>300.0	0.991	0.013	0.036	14.9<br>9.0	104.3<br>99.1
2,4-di-tert butyl phenol	40.0<br>300.0	0.994	0.010	0.027	13.3<br>15.9	99.2<br>103.9
methyl-9,12-octadecadienoate	40.0<br>300.0	0.990	0.014	0,039	8.9<br>11.6	97.9<br>95.9
2,4,6-triphenyl-1-hexene	40.0<br>300.0	0.990	0.011	0.032	12.0<br>13.1	99.4<br>93.4
1,3,5-triphenylcyclohexane	40.0<br>300.0	0.991	0.008	0.025	10.1<br>9.3	96.7<br>99.0
Tetradecanamide	40.0<br>300.0	0.994	0.012	0.034	9.7<br>12.1	100.5<br>84.0
Hexadecanamide	40.0<br>500.0	0.995	0.011	0.031	10.2<br>7.6	99.4<br>94.8
9-Octadecenamide	40.0<br>500.0	0.990	0.020	0.060	5.2<br>4.2	88.3<br>94.6

*mg kg-1 of dry weight sample.

[a] Recovery was assessed in 2 mass fraction levels.
